# BCG vaccination at birth and COVID-19: a case-control study among U.S. military Veterans

**DOI:** 10.1080/21645515.2021.1981084

**Published:** 2021-10-13

**Authors:** Michael N. Bates, Timothy J. Herron, Sandy J. Lwi, Juliana V. Baldo

**Affiliations:** a Research Service, VA Northern California Health Care System, Martinez, CA, USA; b Division of Epidemiology and Biostatistics, School of Public Health, University of California, Berkeley, CA, USA

**Keywords:** BCG, case-control study, COVID-19, off-target effects, retrospective cohort study, SARS-CoV-2, Veterans

## Abstract

In the early stages of the COVID-19 global pandemic, caused by the SARS-CoV-2 virus, low- and middle-income countries (LMICs) appeared to be experiencing lower morbidity and mortality rates than high-income countries, particularly the United States. Various suggestions put forward to account for this included the possibility that LMICs might be experiencing off-target benefits of infant vaccination with BCG, intended primarily to protect against tuberculosis. A number of ecologic epidemiological studies that considered COVID-19 morbidity and mortality rates across countries appeared to support this suggestion. Ecologic studies, however, are primarily hypothesis-generating, given their well-known limitations in extrapolating to the individual-person level. The present study, which employed anonymized records of U.S. Military Veterans treated by the Department of Veterans Affairs was principally a case-control study of COVID-19 infections with a retrospective cohort study of mortality nested within the infections. Controls were a random sample of Veterans not recorded as having had COVID-19. There were 263,039 controls and 167,664 COVID-19 cases, of whom 5,016 died. The combination of country and year of birth was used as a surrogate for infant BCG vaccination. The study did not support the hypothesis that BCG in infancy was protective against COVID-19. The odds ratio for infection was 1.07 (95% confidence interval [CI]: 1.03, 1.11) and the risk ratio for mortality among the COVID-19 cases was 0.86 (95% CI: 0.63, 1.18). The potential for non-differential exposure misclassification was a concern, possibly biasing measures of association toward the null value.

## PLAIN LANGUAGE SUMMARY

- Low- and middle-income countries (LMICs) have appeared to be much less affected by the COVID-19 pandemic, caused by the SARS-CoV-2 virus, than might have been expected from the effects of the virus in more-developed countries. It has been suggested that BCG vaccination of infants against tuberculosis in LMICs might be providing cross-protection against COVID-19. BCG has never been routinely administered in the United States and is not currently administered in most other developed countries.

- Some epidemiology studies, known as “ecologic” studies have provided support for the idea that BCG is protecting against COVID-19. However, ecologic studies, with group (i.e., country) measures of exposure and health outcomes, are difficult to interpret in terms of cause and effect.

- More interpretable are studies that use individual-person measures of exposure and health outcome. We carried out such a study using data from several hundred-thousand U.S. military Veterans, many of whom were born in LMICs and would have received BCG vaccination as infants. Many U.S. Veterans have had COVID-19, and many of those have died of it.
- Our study, the first of its kind, found no evidence to support the idea that infant BCG vaccination protects against infection or death from COVID-19.

## Introduction

The current pandemic of COVID-19 disease caused by the SARS-CoV-2 virus is the most devastating global infectious disease event in 100 years. In the early months of the pandemic, it was widely noted that more-developed countries appeared to be doing much worse, particularly in terms of mortality rates, than low- and middle-income countries (LMICs), including those in Africa and in South Asia. This was especially so for the United States (U.S.), which had the largest number of cases and deaths of any country.

This difference was illustrated by Worldometers data (https://www.worldometers.info/coronavirus/, accessed May 19, 2020), in which the COVID-19 population mortality rate for the U.S. was 280 deaths/million population, while for India it was 2 deaths/million. Most LMICs had COVID-19 mortality rates <20 deaths/million. For India, this suggested a mortality rate ratio estimate, relative to the U.S., of 2/280 = 0.007, and for LMICs, <20/280 or <0.07.

Also, from these data, the COVID-19 incidence rate for the U.S. population was 4,709 cases/million; for India, the corresponding incidence rate was 75 cases/million, and most LMICs had incidence rates <300 cases/million. None of these figures is adjusted for age.

Various suggestions were advanced to account for the differences between high and low-income countries, including that LMICs were at an early stage in their epidemic, they lacked comprehensive national disease surveillance systems, their populations tended to be a lot younger than those of developed countries, and there was a lack of in-country testing capacity for the SARS-CoV-2 virus. These possibilities are likely to account for at least some of the differences, to variable extents across countries. The suggestion additionally arose that these apparently low rates of COVID-19 might be attributable to the fact that virtually all LMICs administer the BCG (Bacillus Calmette–Guerin) vaccine shortly after birth (and in some countries additionally at later ages), for protection against tuberculosis, and sometimes leprosy, as recommended by the World Health Organization.^1,2^ BCG, a live, attenuated vaccine, derived from *Mycobacterium bovis*, has been associated with substantial “off-target” benefits, including reductions in child mortality and protection against some viral diseases.^3^ Although first administered in 1921, BCG has never been universally used in the U.S., nor is it presently administered in most European countries – including those that have experienced major mortality from the pandemic.

A number of epidemiologic studies have explored this hypothesis in relation to COVID-19 morbidity and mortality. Almost all of these have been ecologic studies, which rely on group (i.e., country)-level measures of association. These ecologic studies have fairly consistently shown an apparently protective association between BCG administration at birth and COVID-19 mortality and morbidity,^4–8^ although opinion on these studies has not been unanimous.^9,10^ Ecologic studies have well-recognized limitations in interpretability at the individual-person level, including their inability to adequately account for confounding factors.^11^ There are many differences between LMICs and more developed countries that might account for the differences in COVID-19 morbidity and mortality.

U.S. Veterans served by the U.S. Department of Veterans Affairs (VA) have experienced substantial morbidity and mortality from COVID-19 disease (see: https://www.accesstocare.va.gov/Healthcare/COVID19NationalSummary). The size of this population and the relative uniformity of the electronic healthcare records in the VA system, the largest single healthcare system in the United States, combined with the fact that a substantial proportion of Veterans were born overseas, provided an opportunity to use individual-level data to test the hypothesis of a BCG-related protective effect against COVID-19 morbidity or mortality, unencumbered by the problems of interpreting results of ecologic studies.

The present study is primarily a case-control study of COVID-19 infections, with a retrospective cohort study of case-fatality nested within the cases of infection. Its objective was to examine whether U.S. Veterans born in other countries during a period when national policy was BCG administration for all infants were less likely to be infected with and/or die from the SARS-CoV-2 virus than those who, by reason of age and/or country of birth, would not have been likely to have received a BCG vaccination in infancy. The potential benefit of such a long-lasting protective association with BCG, if real, could potentially extend also to future pandemics, particularly in countries that could not easily obtain or afford the cost of new virus-specific vaccines.^12^

## Methods

This was primarily a case-control study using data, for both male and female veterans, solely from VA electronic health-care records. Before the data were obtained, ethics approval was obtained from the VA Northern California Health Care System Institutional Review Board (IRB). For practical reasons and because the data were anonymized before analysis, informed consent was not obtained from the study subjects, many of whom were deceased. Waivers of informed consent and HIPAA authorization were approved by the IRB. No subject of study was contacted in the course of this research.

### Study subjects

All subjects in the study were required to be alive on January 1, 2020. This requirement was to ensure that anyone deceased because of COVID-19 would be included in the study. The first COVID-19 death in the U.S. occurred on February 28^th^, 2020, in Washington State.

Excluded from both the cases and controls were employees of the VA, who are not necessarily Veterans but may have some occupational health and other records in the system. Excluded also was anyone younger than 20, who were likely to be children of Veterans receiving health care at the VA. Also excluded were Veterans over 75 years of age, as there were very few such older study subjects who would have been likely to have had BCG administered at birth in any country. Excluded also from the analysis of mortality were all persons who were younger than 30 years of age, as deaths from COVID-19 were virtually non-existent in that age group.

Also excluded was anyone with a missing birth date or for whom the country of birth either was not recorded or that country could not be deduced from the recorded city of birth. There were no exclusions from the study based on sex, race or ethnicity.

### Selection of cases

Cases consisted of all Veterans who were recorded as diagnosed with COVID-19 infection in the VA COVID-19 Cohort Master File, as of March 17, 2021. Because there was a delay in bringing records up-to-date, the available data may not have included all Veterans who had been diagnosed at that date. Deaths were indicated in the records, and all deaths occurring within the COVID-19 infection cases have been assumed to be attributable to the virus.

### Selection of controls

Controls consisted of a random sample of 500,000 Veterans recorded as living on January 1, 2020, and who had not been tested for SARS-CoV-2. To compensate for the fact that this sample excluded Veterans who had been tested for the virus, a proportionate sample of those who tested only negative was added into the control sample. The control sample was also obtained on March 17, 2021.

### Data collection

For all cases and controls, we obtained the following key data items: Date of birth, sex, country and city of birth, COVID-19 diagnostic status, and death. Other VA-recorded data, including smoking status and diabetes status, were also obtained for possible investigation as effect modifiers, depending on the main analysis results.

### Exposure

The exposure of interest was being born in a country that had a national policy of administering BCG vaccine to all newborns, during a year in which that policy applied. As a first step, information on country-specific BCG vaccine policies was obtained from the online BCG World Atlas (http://BCGAtlas.org, 2020 update). Because there were gaps in the information available from that source, mainly for the year in which BCG for infants became a national policy, additional information on country-specific BCG policies was obtained by searching the published literature. Supplementary Table S1 provides country-specific BCG data and shows the sources of such information.

Sometimes the country of birth name was not recorded, but the city of birth was. Often it was possible to infer with reasonable confidence the country of birth from the city name. Such country assignment was done blind to COVID-19 status. When it was not possible to make such an inference, no country was recorded, and the subject was not included in the analysis.

Some inferences were made when country-specific BCG data were not available. For example, in the absence of more specific information, former Soviet Union countries (e.g., Kyrgyzstan) were assumed to have applied the same BCG policy as Uzbekistan; in the absence of other information, countries of the former Yugoslavia (e.g., Kosovo) were assumed to have applied the same policy as Bosnia and Herzegovina; countries that were present or recent past territories or dependencies of other countries were assumed to have the same BCG policy as that other country; and some countries known to administer BCG to newborns were assumed to have adopted this practice in 1984, the year that the WHO’s Expanded Program on Immunization added BCG to their standardized vaccination schedule. Anyone born in the United States, including its territories, was assumed not to have received BCG in their birth year, as the U.S. has never had a policy of administering BCG to infants.

Although some countries have administered booster BCG shots at later ages, such as 7 or 14 years, the available information on this was too incomplete to be useful for the data analysis. Similarly, a few countries administered their first BCG shots at a later age (e.g., 13 years). For the purposes of our analysis, subjects born in these countries were considered to be unexposed, as we did not know when they moved to the United States. Some countries had a policy of administering BCG to newborns only in specific groups, such as immigrants from high TB countries or people of a specific ethnic group. Because our focus was on *national* BCG policies, we treated all Veterans born in such countries as unexposed.

All BCG vaccines used around the world derive from the strain developed in France and originally used in 1921. However, over the course of the following century, many BCG substrains, often associated with particular manufacturers, have evolved and been used in different countries. Some countries have administered several different substrains, as their BCG vaccine sources changed. There is some evidence that the different substrains have variable efficacy, at least against TB. Since the quality of information on the use of BCG substrains is unreliable, we did not carry out a BCG substrain-specific analysis.

### Statistical analysis

The initial analysis was descriptive, comparing the distributions of the obtained data elements between cases and controls, separately for both SARS-CoV-2 infection cases and deaths, using Chi-squared tests.

Subsequent analysis of the infection case-control study data involved multivariate unconditional logistic regression, examining whether being born in a country at a time when BCG was part of the infant vaccination policy in that country was associated with reduced odds of being diagnosed with COVID-19 infection. Because it generates risk ratios, appropriate to cohort studies, multivariate log-binomial regression was used to examine the risk of death among those diagnosed with COVID-19 infection.

Possible covariates for confounder adjustment were limited by what was contained within VA records. However, given the primary statistical requirements for confounding to occur, particularly separate associations with both exposure of interest and health outcome, it is difficult to conceive of many possibilities outside the time-variable, age, and some correlates of place of birth, such as race and ethnicity. Sex, although known to be associated with COVID-19 mortality, is not likely to be associated with receipt of BCG vaccination in infancy. We, nonetheless, as a proof of concept, examined sex as a potential confounder of the associations of interest.

In statistical models for infection, age was specified as a categorical variable in 5-year intervals, with the reference category aged 21–25 years; for the mortality analysis, all ages 55 years and below were combined to form the reference category, with the remainder in 5-year categories to age 75. This promoted statistical stability by including an adequate number of deaths in the reference category. Race and ethnicity were used as recorded by the VA, in the standard U.S. Census categories (see Table 1).

**Table 1.** Demographic characteristics of COVID-19 cases and controls, convalescent cases and Covid-19-associated deaths

Characteristic	Case-control study 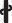		Retrospective cohort study 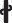	
	Controls (Column %)	Infection cases, including deaths (Column %)	Convalescent infections (Row %)†	Deaths (Row %)†‡
Total	263,039 (100)	167,664 (100)	156,623 (96.9)	5,016 (3.1)
Age (years)				
20–29	9,729 (3.7)	5,931 (3.5)	-	-
30–39	37,337 (14.2)	21,750 (13.0)	21,706 (99.8)	36 (0.2)
40–49	35,318 (13.4)	22,499 (13.4)	22,408 (99.6)	81 (0.4)
50–59	50,624 (19.3)	34,850 (20.8)	34,459 (98.9)	368(1.1)
60–69	63,643 (24.2)	41,184 (24.6)	39,632 (96.3)	1,516 (3.7)
70–75	66,388 (25.2)	41,450 (24.7)	38,418 (92.7)	3,015 (7.3)
Sex				
Male	234,164 (89.0)	147,600 (88.0)	138,001 (96.6)	4,873 (3.4)
Female	28,875 (11.0)	20,064 (12.0)	18,622 (99.2)	143 (0.8)
Race				
White	167,503 (63.7)	107,991 (64.4)	101,077 (96.9)	3,254 (3.1)
American Indian or Alaskan native	2,516 (1.0)	1,943 (1.2)	1,777 (95.2)	89 (4.8)
Asian	3,650 (1.4)	1,957 (1.2)	1,767 (98.1)	35 (1.9)
Black or African American	43,770 (16.6)	41,275 (24.6)	38,897 (96.9)	1,235 (3.1)
Native Hawaiian or other Pacific Is.	2,337 (0.9)	1,870 (1.1)	1,721 (96.8)	56 (3.2)
Unknown/missing	43,263 (16.5)	12,628 (7.5)	11,384 (97.0)	347 (3.0)
Ethnicity				
Not Hispanic or Latino/a	206,387 (78.5)	142,355 (84.4)	133,394 (96.8)	4,366 (3.2)
Hispanic or Latino/a	17,036 (6.5)	18,292 (10.8)	16,361 (97.3)	454 (2.7)
Unknown/missing	39,616 (15.1)	7,806 (4.7)	6,868 (97.2)	196 (2.8)
Marital status				
Married	138,707 (52.7)	86,407 (51.5)	82,113 (96.8)	2,672 (3.2)
Divorced or separated	62,718 (23.8)	47,102 (28.1)	44,787 (96.7)	1,528 (3.3)
Widowed	4,889 (1.9)	3,831 (2.3)	3,576 (93.5)	248 (6.5)
Never married or single	51,337 (19.5)	28,971 (17.3)	24,947 (97.8)	553 (2.2)
Unknown/missing	5,388 (2.1)	1,353 (0.8)	1,200 (98.8)	15 (1.2)
BCG likely in infancy				
No	256,797 (97.6)	162,885 (97.1)	152,168 (96.8)	4,978 (3.2)
Yes	6,239 (2.4)	4,778 (2.9)	4,454 (99.2)	38 (0.8)
Unknown	3 (0)	1 (0)	1 (100)	0 (0)
Diabetes				
Not diagnosed	233,524 (88.8)	128,026 (76.4)	119,026 (97.4)	3,162 (2.6)
Yes	29,515 (11.2)	39,638 (23.6)	37,597 (95.3)	1,854 (4.7)
Hypertension				
Not diagnosed	232,728 (88.5)	131,140 (78.2)	121,740 (97.1)	3,609 (2.9)
Yes	30,311 (11.5)	36,524 (21.8)	34,883 (96.1)	1,407 (3.9)
Smoker				
No	215,466 (81.9)	131,447 (78.4)	122,859 (96.8)	4,062 (3.2)
Yes	47,573 (18.1)	36,217 (21.6)	33,764 (97.3)	954 (2.7)

**
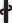
** By χ^2^ test, all associations in this table had *p* ≤ 0.001. † Excludes those aged 20–29 years. ‡ Row % of deaths is the case-fatality rate.

There is the possibility that some Veterans obtained COVID-19 treatment outside the VA and, if so, this could have impacted results. We investigated this possibility by excluding from some analyses Veterans who, based on VA medical department appointment codes, had not had a primary-care-related medical visit at the VA in the last two years.

For diabetes, tobacco smoking and hypertension, effect modification with BCG status on the multiplicative scale wasexamined using both interaction terms and stratified analysis. To test whether there was evidence of a protective effect of BCG that waned with age, we carried out a series of analyses where the upper age limit of subjects was progressively reduced to determine whether any protective association appeared to be increased.

We hypothesized that both the odds ratio (OR) and the mortality risk ratio for being born in a country at a time when BCG was included in that country’s national infant vaccination schedule would be <1.00, indicating protective relationships.

## Results

**Figure 1.**
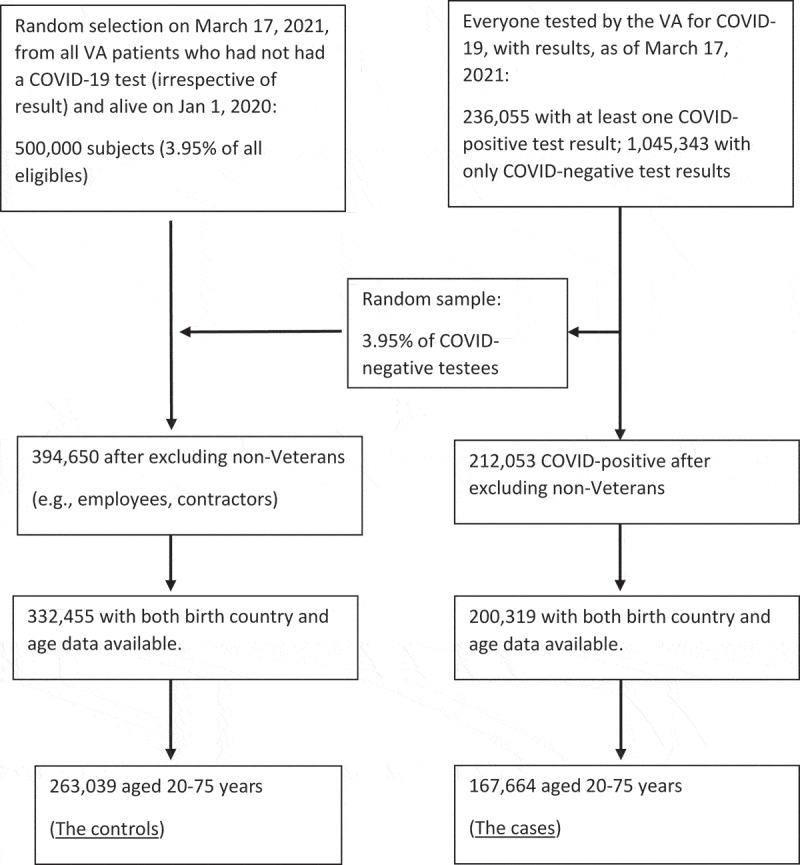
Selection process for VA COVID-19 cases and controls.

The final sample consisted of 430,703 subjects aged 20–75 years, including 167,664 COVID-19 positive cases, of which 161,639 cases were aged 30–75 years, including 5,016 who died. Figure 1 shows the process of reaching the final numbers of study cases and controls, and Table 1 shows the demographics of these groups.

Considering first the infection cases and their controls (columns 2 and 3 in Table 1), it is apparent from the column percentages that there are no more than slight differences between the groups in terms of age and sex. In terms of race, Black and African American people are more prevalent in the case group compared to the controls (24.6% and 16.6%, respectively). However, interpretation is clouded by a higher proportion of controls than infection cases for whom race is unknown or missing (16.5% and 7.5%, respectively).

Turning to mortality, it is clear from the row percentages in column 5 that the case-fatality rate (CFR) increases markedly with age and is higher in males than in females (3.4% and 0.8%, respectively). The latter result is likely a consequence of male COVID-19 cases being considerably older than the female cases. The median and mean for male cases were 61 and 59 years, respectively, with the corresponding figures for females being 50 years for both. With a few exceptions, CFRs are similar across racial and ethnic groups and also by marital status. One exception is the relatively high CFR among widowed subjects (6.5%), likely confounded by older age.

Notably, the CFR in those judged not likely to have had BCG vaccination in infancy (3.2%) is higher than in those considered likely to have had the vaccine (0.8%).

Table 2 shows the results of the case-control study analysis for BCG status and COVID-19 infections using logistic regression. By comparing the ORs adjusted for a single variable with the unadjusted OR (“None” in column 1), it shows the impact on the OR of interest (i.e., for BCG status and COVID-19 infection) from including possible confounders (including sex) in the model one at a time. Each row of the table is a single statistical model. Because inclusion of age, sex or race makes little change to the unadjusted OR, it provides no evidence that any of those variables confounds the main association, but ethnicity does appear to confound it. To investigate further the underlying confounding structure, we carried out an analysis stratified by the two recorded categories of ethnicity status. For the larger category, “Not Hispanic or Latino” (N = 348,081), the OR was 0.97 (95% CI: 0.92, 1.02), and for the category “Hispanic or Latino” (N = 35,197), the corresponding figure was 1.29 (95% CI: 1.20, 1.38).

**Table 2.** Odds ratios and 95% confidence intervals for the association of BCG vaccination in infancy with COVID-19 cases from the US Department of Veterans Affairs, 2020–21, after variable levels of adjustment for potential confounders

Covariates (potential confounders)	Cases	Controls	Odds ratio^†^	95% confidence interval^†^
None	167,663	263,036	1.21	1.16, 1.25
Age	167,663	263,036	1.23	1.18, 1.28
Sex	167,663	263,036	1.20	1.15, 1.25
Race	155,036	219,776	1.22	1.16, 1.27
Ethnicity	159,858	223,423	1.07	1.03, 1.11

† For the association between BCG status and COVID-19 infection. Each row represents a separate statistical model.

We investigated whether restricting controls to those who had their last VA visit within the previous two years substantively affected associations. It moved the odds ratio adjusted for ethnicity in Table 2 further from the null value (OR = 1.13, 95% CI: 1.08, 1.18). When we similarly examined the association for just the Hispanic or Latino category, the OR increased to 1.37 (95% CI: 1.28, 1.48), although for the non-Hispanic or Latino category it remained close to the null value (OR = 1.02, 95% CI: 0.97, 1.08).

Table 3 shows the results of a comparable analysis for mortality within the COVID-19 cases, but with risk ratios (RR) from the log-binomial regression. Similarly to Table 2, the single variable-adjusted RRs for the BCG-Covid-19 mortality association are compared with the corresponding unadjusted RR values.

**Table 3.** Risk ratios and 95% confidence intervals for the association of BCG vaccination in infancy with mortality among COVID-19 cases from the U.S. Department of Veterans Affairs, 2020–21, after variable levels of adjustment for potential confounders

Covariates (potential confounders)	Total COVID-19 infections	Died	Risk ratio^†^	95% confidence interval^†^
None	161,638	5,016	0.27	0.19, 0.37
Age	161,638	5,016	0.86	0.63, 1.18
Sex	161,638	5,016	0.28	0.20, 0.39
Race	149,908	4,669	0.27	0.19, 0.38
Ethnicity	154,575	4,820	0.24	0.17, 0.34

† For the association between BCG status and COVID-19 mortality. Each row represents a separate statistical model.

In contrast to the results in the case–control analysis, age was the major confounder in this analysis. Predictably, as with the infection case–control analysis, sex was not a confounder, but, in contrast to that analysis, ethnicity did not appear to be more than a minor confounder. We consider the result for the BCG-Covid-19 association adjusted by age (RR = 0.86, 95% CI: 0.63, 1.18) to be the most valid for this analysis. When restricted to those study subjects who had a medical appointment with the VA during the last two years, it marginally shifted the age-adjusted RR in Table 3 toward the null value (RR = 0.89; 95% CI: 0.65, 1.22).

To examine the possibility that any BCG protective effect on infection or mortality might have diminished at older ages, we carried out a series of analyses that progressively reduced the upper age of the subjects included in the analyses. This produced no evidence of increased BCG protection against COVID-19 infection or mortality at lower ages (results not shown).

We examined a variety of factors (tobacco use, diabetes, tuberculosis and lung disease) as possible effect modifiers of the relationship between BCG and COVID-19, but none of those analyses provided any evidence of effect modification (results not shown).

## Discussion

This is one of the very few non-ecologic epidemiology studies that has examined the hypotheses that BCG vaccination shortly after birth is responsible for lower COVID-19 infection and/or mortality rates in LMICs. Our study provides no support for either hypothesis.

The only comparable such study of which we are aware involved a regression discontinuity analysis, which found no inflection point in COVID-19 infection and hospitalization rates in birth cohorts that spanned the cessation of BCG administration to Swedish infants in 1975.^13^ According to the BCG World Atlas, Sweden obtained its BCG vaccine from the Danish Statens Serum Institute. Our results are consistent with the Swedish findings, but include people born in LMICs and cover a much wider range of BCG substrains.

In addition to being based on person-level data, our study had considerable strengths compared to the across-country ecologic studies that generated our hypotheses: (1) we were able to adjust results for potential demographic confounders; (2) availability of COVID-19 diagnostic and treatment facilities was fairly uniform across our study subject population; and (3) data were collected in a consistent way within a single large healthcare system. However, before drawing definitive conclusions, it is necessary to consider the study’s possible limitations. The major limitation across both the case-control and the cohort analyses is the potential for exposure misclassification. We had no direct proof that any of the individuals in our study actually did receive BCG vaccination as infants. Such data are not recorded in the VA healthcare system and, indeed, will often be unknown by the individuals themselves. We had no practical option but to infer likely BCG status from the age and country of birth of each of our subjects. We had dates when BCG for infants was introduced as part of the national vaccination policy in many countries, but for other countries we needed to make some assumptions. Apart from that, the official introduction of BCG into a country’s policies does not guarantee that any particular infant will have received it. The rollout of vaccinations after the introduction of a new vaccine policy in a country is often gradual and there may be periodic vaccine supply shortages or other factors that prevent an infant from receiving the vaccine. Additionally, there is some potential for error in the allocation of countries of birth, since some countries were inferred from cities of birth.

The overall result of these uncertainties would have been a non-differential misclassification of exposure status, with a resulting bias in measures of association toward the null value (i.e., 1.00). We do not have information that would allow us to judge the magnitude of any such bias or whether it could have materially affected the conclusions of this study.

Some non-differential misclassification of outcome status, in the form of asymptomatic COVID-19 cases included in the controls for the infection analysis, could also potentially have affected the results of that analysis, again with a bias toward the null. Again, it is not possible to know the extent to which that occurred. This bias could not have been a problem in the mortality analysis as only known infected cases were included.

It is possible that a small number of the deaths among the COVID-19 cases were not caused by the SARS-CoV-2 virus, but that number is unlikely to be more than a small fraction of the deaths and would not substantially affect the results. We did not have available cause of death data.

It is also possible that some of the individuals who comprised our control group could have accessed their medical care outside the VA and been diagnosed with COVID-19 infection in those other facilities. If so, then some cases would inadvertently have been classified as controls in the present study. In the infection analysis that would also have caused a bias toward the null. To investigate this possibility, we restricted some analyses to individuals who had a primary-care-related medical visit with the VA in the last two years, but the results of those analyses, with ORs increasing, provided no evidence that seeking care in non-VA facilities was obscuring a protective effect of BCG.

Although the potential for exposure misclassification was probably the major limitation of our analyses, both for infection and mortality, it is also appropriate to consider the potentials for confounding and selection bias.

There is the possibility that uncontrolled confounding factors (i.e., exposures separately associated with both COVID-19 infection and with being born in a BCG-using country) influenced our results. Our analysis clearly shows that the major confounder of the association between BCG and mortality for which we had data was age (see Table 3). As expected, sex showed no evidence of confounding for either infection or mortality, but ethnicity appeared to confound the odds of infection associated with BCG administration in infancy (Table 2).

The US Census categories for recording of ethnicity data, based around self-identification of Hispanic or Latino, have a long and complicated history (see, for example, https://en.wikipedia.org/wiki/Race_and_ethnicity_in_the_United_States_Census) and are limiting from an epidemiologic perspective. Our stratified analysis of the association of BCG with infection by ethnicity category showed that there was no association with BCG administration among those who identified as non-Hispanic or Latino (OR = 0.97, 95% CI: 0.92, 1.02)), but such an association did exist among those identifying as Hispanic or Latino (OR = 1.29, 95% CI: 1.20, 1.38). This difference suggests that the apparent confounding was largely a consequence of effect modification by ethnicity, but provides no evidence of a protective effect of BCG against infection.

We have few other potential confounder covariates available, but consideration of the prerequisites for confounding suggests that probably few, if any, other factors broadly affecting our study subject population could confound the association between BCG receipts in infancy in countries around the world and, more than 20 years later, infection or death from COVID-19 in the United States. Therefore, on theoretical grounds alone, it is unlikely that there is strong unrecognized confounding that could explain the lack of clear associations between BCG and infection or mortality.

Issues of information bias (mainly non-differential misclassification) and confounding are likely to impact both the case-control and cohort studies in similar ways, but that is not necessarily the case for how selection bias could impact the two epidemiologic study designs. Nonparticipation bias, which can affect all epidemiology studies in which participants are required to provide informed consent, cannot be a problem in either study because the consent of subjects was neither obtained nor necessary. Additionally, there is, the selection bias question that arises in all case–control studies of whether the controls were appropriate to the case group. The standard criterion is that the controls should precisely represent the population that generated the cases. Our control group was a large random sample of all U.S. Veterans who had not tested positive for COVID-19 and cases were all Veterans who had tested positive. These two groups were drawn from exactly the same database and so we have no reason to suspect that the control group was in any way inappropriate to the cases.

Since the cohort analysis of mortality was entirely focused on infection cases and did not include the controls, its results could not have been affected by control selection bias, even if such bias did exist.

In summary, in this, the first study involving people born in many LMICs to examine whether there is a protective effect of BCG administration in infancy against COVID-19 in adulthood, we found no evidence to support or confirm study hypotheses of protection against infection and mortality. However, our results may have been influenced to some extent by misclassification of whether subjects received BCG vaccination in infancy. Such exposure misclassification would have pushed both odds ratios and risk ratios toward the null value, potentially obscuring a protective effect. Further investigation might be undertaken with another large dataset containing a substantial number of COVID-19 cases, but with more definitive data on BCG vaccination status in infancy. Obtaining such a dataset with a balanced distribution of BCG vaccination status by age will generally be difficult for a population born in a single country, as typically all births are subject to that country’s vaccination policy. However, the earlier cited regression discontinuity analysis is one method that could be used more widely for individual countries.^13^

## Supplementary Material

Supplemental Material
