# Supplementary material for: BCG vaccination at birth and COVID-19: a case-control study among U.S. military Veterans
Source: Hum Vaccin Immunother. 2021 Oct 13;18(1):1981084. doi: 10.1080/21645515.2021.1981084 (PMC8986214; doi:10.1080/21645515.2021.1981084)
Supplement: Supplemental Material [file KHVI_A_1981084_SM3671.pdf]

Supplementary Material

BCG Vaccination at Birth and Covid-19: A Case-control Study Among U.S. Military Veterans.

Michael N. Bates[1,2]

Timothy J. Herron[1]

Sandy J. Lwi[1]

Juliana V. Baldo[1]

Affiliations:

VA Northern California Health Care System, Martinez, CA 94553, U.S.A.

Division of Epidemiology, School of Public Health, University of California, Berkeley, CA 94727, U.S.A.

Corresponding author:

Michael N. Bates, Ph.D

VA Northern California Health Care System,

150 Muir Road, Bldg. 26 (mailstop#151)

Martinez, CA 94553, USA.

Tel. +1-510-504-5424

Email. M_bates@berkeley.edu

Table S1. Numbers of Covid-19 infected cases and controls from the VA, by country of birth, with dates of starting and finishing BCG vaccination of infants.

**Country**	**Number of controls**	**Number of infected (cases)**	**BCG for infants**<br>**1 = yes**<br>**0 = no**<br>**. = unknown**	**Year start infant BCG**<br>**(if started)**	**Year infant BCG ended**<br>**(if ended)**	**Source of information†**
Afghanistan	9	6	1	1978		BWA
Albania	5	2	1	1967		BWA
Algeria	5	4	1	1972		BWA
Angola	2	2	1	1979		BWA

Anguilla	2	0	0			UK
Antigua and Barbuda	20	10	1	1954		St Kitts
Argentina	34	23	1	1978		[1]
Armenia	6	7	1	1998		BWA
Aruba	4	1	0			Netherlands
Australia	26	11	1	1955	1985	BWA
Austria	54	18	1	1952	1990	BWA
Azerbaijan	0	1	1	1937		Uzbekistan
Bahamas	19	15	1	1965	1979	Bahamas MoH website
Bahrain	0	1	1	1971	2003	[2]
Bangladesh	7	6	1	1979		BWA
Barbados	40	25	1	1956	2016	BWA
Belarus	3	2	1	1937		Uzbekistan
Belgium	29	9	0			BWA
Belize	69	40	1	1953		BWA
Benin	5	4	1	1982		[3]
Bermuda	16	11	0			UK
Bolivia	16	13	1	1975		[4]
Bosnia and Herzegovina	8	7	1	1950		BWA
Brazil	55	42	1	1976		BWA
Brunei	1	0	1	1984		EPI/WHO
Bulgaria	7	3	1	1951		BWA
Burkina Faso	1	1	1	1984		[5]
Cambodia	21	19	1	1986		BWA
Cameroon	16	17	1	1976		BWA
Canada	605	321	0			BWA

Cape Verde	6	11	1	1956		[6]
Cayman Islands	1	0	0			UK
Chile	39	47	1	1953		BWA
China	185	47	1	1949		BWA
Colombia	233	235	1	1960		BWA
Congo, Dem Rep	4	3	1	1984		BWA
Congo, Rep	3	1	1	1984		EPI/WHO
Costa Rica	27	21	1	1976		[4]
Cote d'Ivoire	11	7	1	1978		BWA
Croatia	5	2	1	1948		BWA
Cuba	227	202	1	1962		BWA
Curaçao	1	2	0			Netherlands
Cyprus	1	0	0			[7]
Czech Republic	11	5	1	1953		BWA
Denmark	9	3	1	1946	1986	BWA
Dominica	31	16	1	1954		St Kitts
Dominican Republic	195	239	1	1975		[4]
Ecuador	109	171	1	1978		[4]
Egypt	24	22	1	1974		[8]
El Salvador	119	145	1	1970		BWA
Equatorial Guinea	1	0	1	1984		EPI/WHO
Eritrea	1	2	1	1981		Ethiopia
Estonia	2	0	1	1948		BWA
Ethiopia	23	19	1	1981		BWA
Fiji	11	8	1	1965		BWA

Finland	4	1	1	1941	2006	BWA
France	125	68	1	1950	2007	BWA
Gambia, The	2	3	1	1979		BWA
Georgia	2	0	1	1979		BWA
Germany	1,790	943	1	1953	1998	BWA
Ghana	63	62	1	1978		BWA
Gibraltar	0	1	0			UK
Greece	44	30	1	1949	2016	[9]
Greenland	1	0	1	1955		BWA
Grenada	25	11	1	1954		St Kitts
Guatemala	91	85	1	1980		[10]
Guadeloupe	0	1	1	1950	2007	France
Guinea	4	2	1	1984		EPI/WHO
Guyana	117	96	1	1955		BWA
Haiti	183	176	1	1992		BWA
Honduras	83	88	1	1954		BWA
Hungary	22	8	1	1953		BWA
Iceland	11	7	0			[11]
India	69	48	1	1962		BWA
Indonesia	22	17	1	1977		BWA
Iran	27	16	1	1984		BWA
Iraq	20	21	1	1985		[12]
Ireland	30	19	1	1949	2015	BWA
Israel	20	10	1	1955	1982	BWA
Italy	199	120	0			BWA
Jamaica	423	285	1	1978		BWA
Japan	389	187	1	1951		BWA
Jordan	3	6	1	1949		BWA

Kazakhstan	2	2	1	1965		BWA
Kenya	33	30	1	1985		BWA
Korea, South	424	142	1	1960		BWA
Kosovo	0	2	1	1950		Bosnia & Herzegovina
Kuwait	5	0	1	1962		BWA
Kyrgyzstan	5	1	1	1937		Uzbekistan
Laos	35	20	1	1982		[13]
Latvia	6	1	1	1962		BWA
Lebanon	17	18	0			[14]
Lesotho	1	1	1	1984		EPI/WHO
Liberia	33	36	1	1984		EPI/WHO
Libya	13	1	1	1971		[15]
Lithuania	2	2	1	1962		[16]
Luxembourg	2	1	1	1950	2007	France
Malawi	2	0	1	1977		[17]
Malaysia	6	4	1	1961		BWA
Mali	0	3	1	1961		BWA
Malta	2	3	1	1950		BWA
Marshall Islands	13	11	1	2004		BWA
Mauritania	0	1	1	1984		EPI/WHO
Mauritius	1	0	1	1984		EPI/WHO
Mexico	1,263	1,568	1	1951		BWA
Micronesia, Fed States	25	21	1	1989		[18]
Moldova	6	3	1	1926		BWA
Mongolia	1	0	1	1948		BWA
Montserrat	1	0	0			UK

Morocco	36	29	1	1967		BWA
Mozambique	1	0	1	1984		EPI/WHO
Myanmar	4	3	1	1986		[19]
Namibia	3	0	1	1984		EPI/WHO
Nepal	25	20	1	1979		BWA
Netherlands	54	23	0			BWA
New Zealand	7	5	0			BWA
Nicaragua	84	97	1	1976		[4]
Niger	16	12	1	1979		BWA
Nigeria	61	52	1	1991		BWA
North Macedonia	0	2	1	1948		BWA
Northern Marianas	37	14	0			USA
Norway	11	5	1	1947	2009	BWA
Pakistan	27	21	1	1970		BWA
Palau	17	15	0			USA
Panama	289	214	1	1977		[4]
Papua New Guinea	2	0	.	1974		
Paraguay	3	4	1	1976		[4]
Peru	130	171	1	1962		BWA
Philippines	1,893	1,027	1	1979		BWA
Poland	70	26	1	1955		BWA
Portugal	40	30	1	1965	2016	BWA
Romania	34	15	1	1951		Bulgaria
Russia	45	19	1	1937		Uzbekistan
St Kitts and Nevis	19	5	1	1953		BWA

St Lucia	17	6	1	1954		St Kitts
St Vincent & the Grenadines	7	7	1	1954		St Kitts
Samoa	15	19	1	1966		[20]
San Marino	1	1	.			
Saudi Arabia	11	7	1	1979		BWA
Senegal	5	2	1	1986		BWA
Serbia	11	6	1	1950		[21]
Seychelles	2	0	1	1984		EPI/WHO
Sierra Leone	14	12	1	1990		BWA
Singapore	3	3	1	1955		BWA
Slovakia	2	0	1	1953	2012	BWA
Slovenia	1	0	1	1947	2005	BWA
Somalia	5	2	1	1984		EPI/WHO
South Africa	11	4	1	1973		BWA
South Sudan	0	4	1	1976		Sudan
Spain	90	61	1	1965	1981	BWA
Sri Lanka	2	1	1	1963		BWA
Sudan	9	7	1	1976		BWA
Suriname	3	1	.			
Sweden	12	3	1	1940	1975	BWA
Switzerland	7	6	0			
Syria	4	5	1	1985		Iraq
Taiwan	51	20	1	1965		BWA
Tajikistan	2	0	1	1937		Uzbekistan
Tanzania	2	3	1	1975		BWA
Thailand	92	84	1	1977		BWA

Togo	10	4	1	1980		[22]
Tonga	3	4	1	1969		[20]
Trinidad and Tobago	195	119	1	1952	1976	BWA
Tunisia	3	4	1	1979		BWA
Turkey	21	18	1	1951		BWA
Turkmenistan	0	2	1	1937		Uzbekistan
Turks and Caicos	1	0	0			UK
Uganda	9	4	1	1987		BWA
UAE	2	0	1	1986		[23]
UK	532	256	0			BWA
Ukraine	39	14	1	1962		BWA
Uruguay	2	1	1	1980		BWA
USA	250,650	158,980	0			BWA
Uzbekistan	10	4	1	1937		BWA
Venezuela	37	34	1	1976		[4]
Vietnam	199	128	1	1985		BWA
Virgin Is British	6	3	0			UK
Yemen	1	3	1	1984		EPI/WHO
Zimbabwe	1	2	1	1966		[24]
TOTAL	263,039	167,664				

† BWA = BCG World Atlas (http://bcgatlas.org/index.php); Square brackets [ ] = Reference; EPI/WHO = 1984, the year the Expanded Program on Immunization of the World Health Organization added infant BCG immunization to its schedule; Country name = Other country whose BCG policy was assumed to have been adopted.

References

[1] Miceli I, de Kantor IN, Colaiácovo D, Peluffo G, Cutillo I, Gorra R, et al. Evaluation of the effectiveness of BCG vaccination using the case-control method in Buenos Aires, Argentina. Int J Epidemiol 1988;17:629–34. https://doi.org/10.1093/ije/17.3.629.

[2] Malik SK, Khalfan S. The epidemiology of tuberculosis in Bahrain. Tubercle 1990;71:51–4. https://doi.org/10.1016/0041-3879(90)90061-c.

[3] Nackers F, Dramaix M, Johnson RC, Zinsou C, Robert A, DE Biurrun Bakedano E, et al. BCG vaccine effectiveness against Buruli ulcer: a case-control study in Benin. Am J Trop Med Hyg 2006;75:768–74.

[4] Ochoa LC. Introducing the EPI Newsletter. EPI Newsl 1 1 May 1979 1979.

[5] Ouédraogo N, Kagoné M, Sié A, Becher H, Müller O. Immunization coverage in young children: a study nested into a health and demographic surveillance system in Burkina Faso. J Trop Pediatr 2013;59:187–94. https://doi.org/10.1093/tropej/fms075.

[6] Sarmentó A. Trends in Mortality in the Cape Verde Islands. An Inst Med Trop (Lisb) 1959;16:229–66.

[7] Sakurada K, Toida I, Sakai I, Sekiguchi K, Shiraishi T, Takatori T. The BCG scar after percutaneous multiple puncture vaccination may help establish the nationalities of unidentified cadavers. J Clin Forensic Med 2003;10:235–41. https://doi.org/10.1016/j.jcfm.2003.08.004.

[8] Madkour M, Khalifa AS. A critical review of B.C.G. vaccination programme in Egypt. J Trop Med Hyg 1977;80:144–6.

[9] Cherry RL, Mangun CW. Technical assistance in public health; the 6 year program in Greece. Public Health Rep Wash DC 1896 1954;69:475–86.

[10] Burkhalter BR, Miller RI, Silva L, Burleigh E. Variations in estimates of Guatemalan infant mortality, vaccination coverage, and ORS use reported by different sources. Bull Pan Am Health Organ 1995;29:1–24.

[11] Szigeti R, Kellermayer D, Trakimas G, Kellermayer R. BCG epidemiology supports its protection against COVID-19? A word of caution. PloS One 2020;15:e0240203. https://doi.org/10.1371/journal.pone.0240203.

[12] al-Sheikh OG, al-Samarrai JI, al-Sumaidaie MM, Mohammad SA, al-Dujaily AA. Immunization coverage among children born between 1989 and 1994 in Saladdin Governorate, Iraq. East Mediterr Health J Rev Sante Mediterr Orient Al-Majallah Al-Sihhiyah Li-Sharq Al-Mutawassit 1999;5:933–40.

[13] Arnadottir TH, Soukaseum H, Vangvichit P, Bounmala S, Vos E. Prevalence and annual risk of tuberculosis infection in Laos. Int J Tuberc Lung Dis Off J Int Union Tuberc Lung Dis 2001;5:391–9.

[14] Miller A, Reandelar MJ, Fasciglione K, Roumenova V, Li Y, Otazu GH. Correlation between universal BCG vaccination policy and reduced mortality for COVID-19. MedRxiv 2020.

[15] Rayes AA, Annajar BB, Dayhum AS, Eldaghayes IM. Why there were few cases of coronavirus disease 2019 in Libya during the first two months of the pandemic? Int J One Health 2020;6:160–4.

[16] Suciliene E, Rønne T, Plesner AM, Semenaite B, Slapkauskaite D, Larsen SO, et al. Infant BCG vaccination study in Lithuania. Int J Tuberc Lung Dis Off J Int Union Tuberc Lung Dis 1999;3:956–61.

[17] Fine PE, Ponnighaus JM, Maine N. The distribution and implications of BCG scars in northern Malawi. Bull World Health Organ 1989;67:35–42.

[18] Lambert ML. Foreign aid and tuberculosis control policy in the Federated States of Micronesia. Lancet Lond Engl 1996;347:334–5.

[19] Myint TT, Win H, Aye HH, Kyaw-Mint TO. Case-control study on evaluation of BCG vaccination of newborn in Rangoon, Burma. Ann Trop Paediatr 1987;7:159–66. https://doi.org/10.1080/02724936.1987.11748499.

[20] Organization WH. Tuberculosis control in the Western Pacific Region: 1951-1970. Manila: WHO Regional Office for the Western Pacific; 1972.

[21] Simeonov LA, Jarec Z. Skin sensitivity of schoolchildren in Yugoslavia to different mycobacterial sensitins. Bull World Health Organ 1971;45:657–66.

[22] Phillips RO, Phanzu DM, Beissner M, Badziklou K, Luzolo EK, Sarfo FS, et al. Effectiveness of routine BCG vaccination on buruli ulcer disease: a case-control study in the Democratic Republic of Congo, Ghana and Togo. PLoS Negl Trop Dis 2015;9:e3457. https://doi.org/10.1371/journal.pntd.0003457.

[23] Sedaghatian MR, Shana'a IA. Evaluation of BCG at birth in the United Arab Emirates. Tubercle 1990;71:177–80. https://doi.org/10.1016/0041-3879(90)90072-g.

[24] Cranefield PF. Philately, public health education and medical history: The 1982 Zimbabwe stamps commemorating the discovery of the tubercle bacillus. Bull N Y Acad Med 1986;62:206–8.
